# Therapeutic Carbohydrate Restriction as a Metabolic Modality for the Prevention and Treatment of Abnormal Uterine Bleeding

**DOI:** 10.3390/nu15173760

**Published:** 2023-08-28

**Authors:** Andrea C. Salcedo, Jane Yun, Cody Carter, Elaine Hart

**Affiliations:** 1 Department of Gynecology and Obstetrics, School of Medicine, Loma Linda University, Loma Linda, CA 92354, USA; 2 Department of Pathology and Human Anatomy, School of Medicine, Loma Linda University, Loma Linda, CA 92354, USA

**Keywords:** insulin resistance, cardiovascular disease, metabolic syndrome, therapeutic carbohydrate restriction, AUB, PCOS, leiomyoma, endometrial polyps

## Abstract

Therapeutic carbohydrate restriction diets have been becoming increasingly popular over the years, resulting in dramatic weight loss and an improvement in metabolic disorders. Obesity, insulin resistance, and diabetes are the risk factors for many gynecologic morbidities such as uterine leiomyoma, endometrial polyps, and polycystic ovarian syndrome. There is evidence suggesting that the pathogenesis of cardiovascular disease is similar to that seen in many causes of abnormal uterine bleeding. We aim to explain how cardiovascular disease risk factor reduction with the use of therapeutic carbohydrate restriction may prevent and potentially treat these gynecologic disorders.

## 1. Introduction

Abnormal uterine bleeding (AUB) is the most common reason for gynecologic consultation in the United States, resulting in USD 34 billion of health care costs annually [1,2]. One in three women will have undergone a hysterectomy by the age of 60, resulting in high health care utilization to treat this problem [3]. National statistics reveal that the use of inpatient hysterectomy has decreased over the years [3]. However, the total number of hysterectomies performed are likely under-reported since most hysterectomies are performed in the outpatient setting [4]. This results in an underestimation of approximately 100,000–200,000 cases annually [4]. Despite medical and surgical options to treat AUB, the impact of this disorder continues to be a burden on the quality of life and results in an increased utilization of health care resources [5]. As such, while the health care system addresses the effects of abnormal uterine bleeding, focus on the practical application and early prevention of the development of this disease has been lacking. There is a broad knowledge base of the pathophysiologic similarities in the development of both structural and nonstructural causes of AUB and cardiovascular disease [6,7,8,9,10,11]. Yet, there is little focus on the prevention of cardiovascular disease risk factors to reduce the index risk and treatment of AUB, outside of polycystic ovarian syndrome (PCOS). A review of the current literature indicates that there are well-established risk factors associating hypertension, obesity, and metabolic syndrome with leiomyoma [12,13,14,15,16]. There is also documented evidence in the literature regarding the similar pathologic development of leiomyoma with atherosclerotic disease [6,10,13,17]. Similarly, metabolic risk factors, such as insulin resistance and obesity, and inflammatory factors are associated with the development of endometrial polyps [18,19,20]. First-line therapy for reducing the risk of cardiovascular disease in polycystic ovarian syndrome includes exercise and weight loss, yet no specific regimen is supported [21].

Treatment strategies pertaining to uterine leiomyomata and endometrial polyps primarily involve surgical intervention. When examining the pathophysiology of the development of these disorders, they can be attributed to the effects of insulin resistance, the development of smooth muscle endothelial inflammation, or both [6,10,15,20,22,23]. With this understanding, clinicians can make connections between similar pathologic developments between cardiovascular disease and the prominent causes of abnormal uterine bleeding, particularly leiomyomata, endometrial polyps, and endometrial and ovulatory dysfunction. The purpose of this opinion is to explain the shared pathophysiology of cardiovascular and metabolic diseases with the common causes of abnormal uterine bleeding. With this understanding, we propose how lifestyle strategies to reduce cardiovascular risk should apply to the prevention and treatment of AUB.

In this clinical opinion, we first compare the pathways in which the structural and ovulatory causes of AUB share pathophysiology with cardiovascular disorders. Second, we discuss the main mechanisms in which therapeutic carbohydrate restriction (TCR) treats metabolic disease. Then, we discuss how TCR is a preventive measure and therapeutic modality in managing these common gynecologic disorders. 

## 2. Systemic Endothelial Inflammation Leading to Structural Causes of AUB-Leiomyomata, Endometrial Polyps, and Endometrial Dysfunction

It is well established that smooth muscle endothelial inflammation leads to cardiovascular end-organ disease. This pathologic change in the microcirculation can lead to end-organ damage in all vascularized organs [24]. Examples of end-organ-specific injuries include myocardial infarction, chronic kidney disease, non-alcoholic fatty liver disease, and diabetic retinopathy [24,25]. 

Many causes of AUB come from similar pathophysiological origins. AUB is classified by uterine structural (e.g., endometrial polyps, adenomyosis, leiomyoma, and malignancy and hyperplasia) and nonstructural causes (e.g., coagulopathy, endometrial, ovulatory, iatrogenic, and not otherwise specified causes), known as PALM-COEIN, adopted by the International Federation of Gynecology in 2011 [26]. The classification of AUB is helpful in assigning treatment modalities once the disease process is diagnosed. Endometrial/endocervical polyps (AUB-P), leiomyoma (AUB-L), adenomyosis (AUB-A), and ovulatory dysfunction (AUB-O) are the most common causes of menstrual irregularities among reproductive-age women [26,27]. When examining the pathophysiology of the development of these disorders, they can be attributed to the effects of insulin resistance, the development of smooth muscle endothelial inflammation, or both.

**Figure 1:**
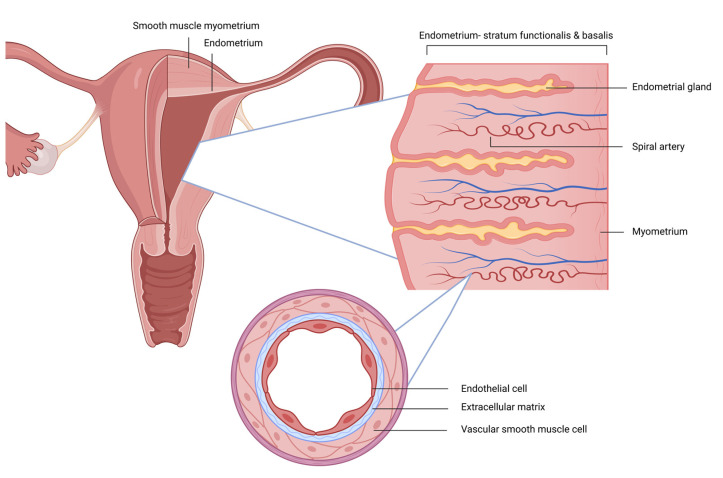
The uterus: a smooth muscle structure among smooth muscle arteries.

The uterus can be thought of as an end organ. It is anatomically and histologically a matrix of arterial smooth muscle (Figure 1). The endometrium is a cyclically regenerating and degenerating bed of glandular cells vascularized by the basal and spiral arteries, with the latter extending into the stratum functionalis layer of the endometrium. With progesterone withdrawal and its anti-inflammatory effect, there is an increase in local cytokines, the infiltration of leukocytes into the endometrium, and spiral artery constriction with resultant menses [28]. These vascular beds are prone to similar endothelial dysfunction associated with insulin resistance and metabolic dysfunction, as seen in other areas of the body. In 2022, Salcedo et al. demonstrated an elevated prevalence of insulin resistance among women with abnormal uterine bleeding [29]. The study also demonstrated that body mass index and an increased waist/hip ratio are independent predictors of insulin resistance among women with abnormal uterine bleeding [29]. 

**Figure 2:**
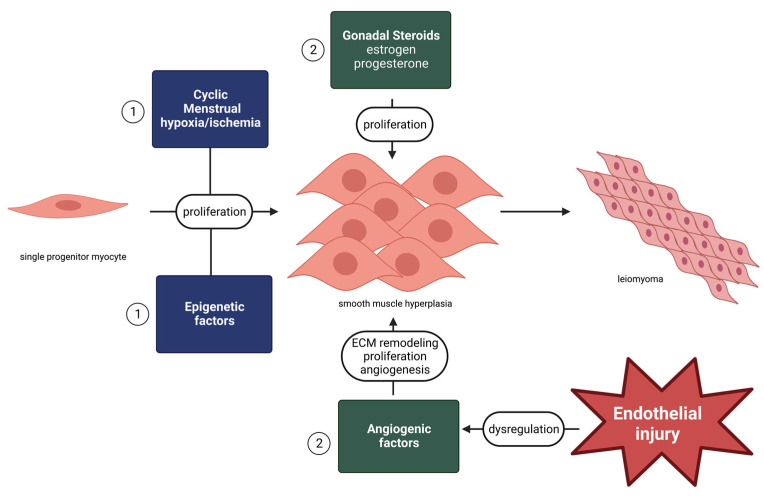
Two-step leiomyoma development, influenced by endothelial injury.

Within the uterine corpus specifically, uterine leiomyoma formation results from at least two steps; the first step is the conversion of a single normal progenitor myocyte into an abnormal myocyte triggered by epigenetic factors that alter genetic signaling through a variety of mechanisms such as point mutations, chromosomal abnormalities, and heritable genetic susceptibility [16]. This is followed by the stimulation of the growth of the progenitor myocyte via gonadal steroid activation, and the abnormal activation of angiogenic growth factors (Figure 2) [7,8,16]. Menstrual hypoxic and ischemic events at the level of the endometrium lead to genetic aberrations that result in smooth muscle proliferation, which leads to myometrial hyperplasia [16,30]. Endothelial injury leads to the pathologic activation of angiogenic growth factors, such as the vascular endothelial growth factor (VEGF), fibroblast growth factor (FGF), epidermal growth factor (EGF), and transforming growth factor (TGF-β), which leads to hyperproliferation, angiogenesis, and extracellular matrix deposition, resulting in uterine leiomyoma [7,31]. 

By understanding these associations, one may parallel the pathophysiology with what is known in the development of cardiovascular disease. Looking back to one of the initial observations comparing leiomyoma development with atherosclerotic disease, research continues to correlate these findings. The monoclonal origination of leiomyoma was postulated to be similar to atherosclerotic plaques, as seen with endothelial irritation from hypertension and a higher risk of uterine fibroids [17]. Obesity is characterized by a chronic state of inflammation and the secretion of adipokines; these adipokine elevations are seen in the development of leiomyoma [32,33]. Dysregulation in the angiogenic growth factors, such as EGF, TGF-β, and VEGF, are associated with uterine fibroid development [7,8]. Finally, increased levels of inflammatory cytokines, including interleukin (IL-1, IL-6) and tumor necrosis factor α (TNF-α), as well as increased numbers of reparative macrophages, were found to be present within leiomyoma [34]. This is exemplified by Photomicrograph 1. Protic et al. (2016) noted that uterine leiomyomas were found to have a significantly higher number of macrophages in the nearby myometrium in comparison to distant myometrium away from the tumor [34]. In the cellular subtype of leiomyomata, there was an increase in both macrophages and mast cells [34]. 

Although the pathophysiology of the development of endometrial polyps is largely unknown, two main pathways were postulated—estrogen-dependent stimulus [18] and chronic inflammation [23,35,36]. First, increased levels of endogenous and exogenous unopposed estrogen seem to be associated with the development of endometrial polyps [18]. It is known that endometrial polyp glandular epithelial cells possess increased ratios of estrogen to progesterone receptors compared to those of normal endometrial glandular cells of the stratum functionalis [18]. As such, elevated concentrations of estrogen will stimulate the endometrium to favor this ratio and result in the conversion of endometrial glands to polyp tissue [18]. Endogenously, the aromatase enzyme converts androgens into estrogens in the adipose [18]. Excess levels of estrogen secreted by the subcutaneous adipose tissue stimulate the endometrium to favor polyp production. The upregulation of aromatase, insulin, cortisol, xeno-estrogens, free fatty acids, inflammatory cytokines, and estradiol itself collectively upregulate the activity of aromatase to effectively modify testosterone levels and increase the intracellular concentration of estradiol [37,38]. Exogenous sources of estrogen receptor stimulation in the endometrium, such as the selective estrogen receptor modulator tamoxifen, which is used in the treatment of breast cancer, have been associated with the development of endometrial polyps [18]. Like the development of uterine leiomyoma, there also appears to be a genetic component to the development of endometrial polyps with the identification of three chromosomal translocations that are seen in higher frequencies with these lesions, for a total of four genotypes (including normal genotypes) [18].

Second, the role of chronic inflammation in the pathophysiology of endometrial polyps is supported by studies that performed an interrogation of their immune cellular environment. El-Harmaneh et al. (2013) noted that tryptase-, chymase-, and c-KIT-expressing mast cells were seen in higher concentrations in endometrial polyps and in adjacent and distant endometrium when compared to control endometrium in patients without endometrial polyps [36]. This supports the hypothesis that endometrial polyps represent an inflammatory lesion [36]. Tryptase and chymase are secreted by mast cells, and c-KIT receptors are activated to stimulate endothelial cell proliferation and extracellular matrix degradation, which contribute to angiogenesis [39,40]. Further, T regulatory cells were similarly also seen in higher levels compared to normal functional endometrium [36].

Excess levels of reactive oxygen species and mast cells interfere with the normal development of the endometrial lining [23,35]. Through the activation of the inflammatory cascade, excessive mast cell presence leads to the activation of angiogenesis within the endometrium and increases the blood vessel density [18]. Higher levels of angiogenic growth factors such as VEGF and TGF-β as well as mast cell activation have been found to be present in nasal and colorectal polyps, in addition to endometrial polyps, suggesting that inflammatory cytokines are likely a common etiologic factor to each of these growth disorders as well [23,40,41]. Not surprisingly, the development of colorectal adenomatous polyps shared similar risk factors as those for endometrial polyps with an increased BMI, abdominal obesity, and a sedentary lifestyle [41,42,43].

## 3. Insulin Resistance in Ovulatory Dysfunction

A fundamental concept of irregular menses in reproductive-age women is based on the dysregulation of insulin levels, which is seen in polycystic ovarian syndrome (PCOS) [44,45]. A hyperinsulinemic state leads to the stimulation of the luteal cells of the ovary to produce excessive levels of androgens in concert with decreasing sex-hormone-binding globulin production by the liver, which results in the net effect of excessive circulating androgen concentrations [46,47]. These elevations in insulin may also stimulate the adipose tissue to produce elevations in androgens as well [28,48]. The consequent hyperandrogenemia results in the inhibition of the GnRH pulsatory index at the level of the hypothalamus, which inhibits normal ovulatory gonadotropin signaling at the level of the anterior pituitary [44,47]. As the ovulatory cycle is interrupted, the excess levels of estrogen and the lack of ovulatory progesterone lead to the hyperproliferation of the stratum functionalis layer of the endometrium [44,47]. Eventually, the hyperproliferation of the functional endometrium will outgrow its vascular supply and lead to unscheduled menstrual shedding that results in AUB [30,49]. This is commonly reported as disordered proliferative endometrium with glandular and stromal breakdown changes when seen via pathologic evaluation.

## 4. Mechanisms in Which Therapeutic Carbohydrate Restriction Treats Metabolic Diseases

Carbohydrate-restricted diets were the mainstay treatment of metabolic diseases, particularly type 2 diabetes [50,51]. Yet, current conventional treatment strategies primarily center on the achievement of normoglycemia through pharmaceuticals [50,51]. After the development of the lipid hypothesis [52,53,54], the treatment paradigm quickly shifted to a low-fat model of nutrition, and consequently, a higher carbohydrate treatment regimen.

Acknowledging the multifactorial pathogenesis to the development of atherosclerotic disease proposed by Davignon in 1978 [54], the treatment of metabolic disease through the reduction in insulin resistance is in order [50,55]. Understanding metabolic syndrome as a preamble to the development of cardiovascular disease, which is an approach that keeps the five diagnostic criteria (elevated waist/hip ratio, low HDL, elevated triglycerides, elevation in blood pressure, and hyperglycemia) in mind, will thereby reduce overall risk [55]. A review of the literature regarding carbohydrate restriction reveals a successful therapeutic approach in the treatment of metabolic disease without the use of medical or surgical intervention [50,51,55]. Furthermore, a systemic review also supports the notion that low-carbohydrate diets may be similar to or somewhat superior to low-fat diets in the treatment of type 2 diabetes [56]. Other studies indicate that, while balancing the LDL and cholesterol elevations, low-carbohydrate diets improve metabolic markers such as triglycerides, HDL, and weight [57,58].

The biochemical basis for this concept is that the reduction in carbohydrates as an energy source leads to a compensatory increase in fat metabolism as a physiologic alternative [59]. Intermittent fasting or the decreased consumption of carbohydrates (<50 g/day) leads the body to utilize stored fuel sources such as fat and glycogen [59]. This process of gluconeogenesis and ketogenesis in the liver is stimulated by glucagon and low levels of insulin [60,61]. The release of non-esterified fatty acids and the production of ketone bodies can be used as an alternative substrate for the tricarboxylic acid (TCA) cycle, which leads to higher proportions of adenosine triphosphate (ATP) production per substrate molecule compared to glucose metabolism [59]. This has the added benefit of using a high energy substrate that also reduces lipid stores [59]. Therefore, the switch from carbohydrate metabolism to ketone metabolism leads to a reduction in triglyceride concentrations, elevations in high-density lipoproteins (HDL), and reductions in atherogenic inflammatory markers [55,59]. Furthermore, reductions in the consumption of carbohydrates result in decreased levels of insulin and improvements in insulin resistance, which thereby improves glycemic control in type 2 diabetics and PCOS [46,50,51,59].

Various organizational bodies recommend the reduction in added and refined sugars as a method to reduce the cardiovascular disease risk, while permitting higher-quality carbohydrate sources such as legumes and whole grains [62,63]. While the approach to treating metabolic disease is still under considerable debate, a treatment methodology that involves reducing insulin resistance is in line with the established management of PCOS. This treatment strategy among women with PCOS could reduce the risk of the subsequent development of leiomyoma, which is an associated risk factor [64].

## 5. How Therapeutic Carbohydrate Restriction Can Be a Preventive and Treatment Modality in Managing These Common Gynecologic Disorders

A recent review highlights the role that carbohydrate-restricted diets can have in reducing common endocrine diseases of the female reproductive system, including infertility, PCOS, and endometrial cancer [65]. The chronic circulation of pro-inflammatory molecules leads to organ-specific injuries that are the hallmark of hyperinsulinemia and insulin resistance. Reductions in carbohydrate consumption can similarly lead to reductions in inflammatory markers and dysregulation in the hypothalamic–pituitary–ovulatory axis. Applying the same concepts to the reduction in atherogenic disease, reductions in inflammatory markers may thereby similarly reduce the pathologic development of smooth muscle angiogenesis seen in the development of uterine leiomyomata and endometrial polyps. Translating the compelling evidence that TCR diets can address aberrant metabolic processes contributing to endothelial dysfunction and angiogenesis, discrete lifestyle treatments may prevent, improve, or treat the origins of these gynecologic disorders. By applying the understanding of the sources of AUB in the PALM-COEIN approach [26], primary and secondary prevention to reduce the eventual development of gynecologic disease would lead to a reduction in disease burden. However, outside of the treatment for PCOS, rigorous studies testing TCR diets with other lifestyle interventions for the prevention and treatment of these common and costly entities are lacking [46]. This leads to two main targets for investigative discovery within gynecology: metabolic reduction in inflammatory markers that lead to structural anomalies in AUB, and focused, nutritional, and endocrinologic treatment of ovulatory disorders, such as PCOS. The reduction in smooth muscle inflammation and chronic inflammatory factors may prevent or slow the disease progression seen in the structural causes of AUB. TCR may offer a directive approach in the lifestyle treatment of PCOS, which is the recommended first-line treatment strategy according to the American College of Obstetricians and Gynecologists [21].

## 6. Conclusions

While standard medical and surgical treatments are valuable strategies, a lifestyle-based intervention, such as therapeutic carbohydrate restriction, may prevent and treat abnormal uterine bleeding. International organizations rely on medical and surgical management for the treatment of gynecologic diseases that lead to abnormal uterine bleeding [26,27]. Furthermore, clinical guidelines and evidence suggest lifestyle intervention and the use of hypoglycemic medications as first-line treatments for polycystic ovarian syndrome [21,66]. Evidence supporting therapeutic carbohydrate restriction as a treatment modality for the reduction in cardiovascular disease and the reversal of type 2 diabetes is available. The reduction in hyperglycemia will thereby lead to less end-organ damage. The uterus can be considered a recipient of end-organ damage. Given the overlap in the pathophysiologic mechanisms of atherogenic vascular disease and the common causes of abnormal uterine bleeding, the same principles for reducing the overall burden of gynecologic disease should be considered and further studied. Based on this evidence-supported hypothesis, we strongly recommend clinical studies of insulin resistance bloodwork parameters in patients with abnormal uterine bleeding. We also present a rationale for a testable hypothesis of therapeutic carbohydrate restriction as a treatment option to be trialed clinically. This can lead to a new recommendation of therapeutic carbohydrate restriction as a preventive and therapeutic lifestyle intervention in selected patients with associated causes of abnormal uterine bleeding.

## Figures and Tables

**Photomicrograph 1.**
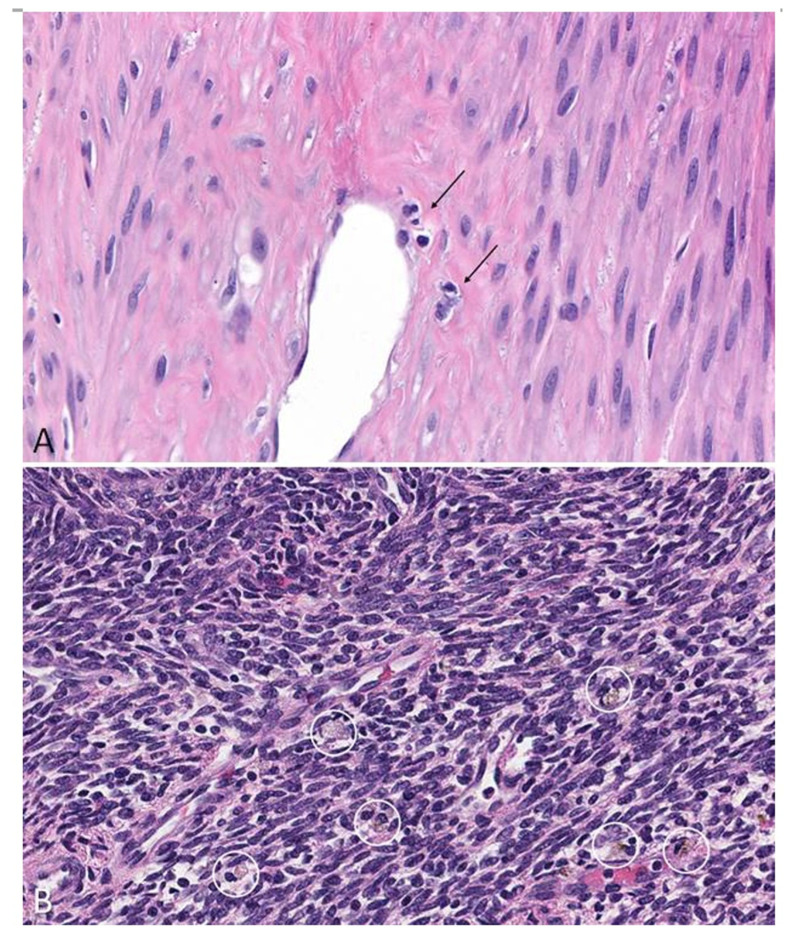
Photomicrographs of macrophages within leiomyomata. (**A**): Conventional leiomyoma with small collection of perivascular macrophages (arrows; 400× magnification, H&E stain). (**B**): Cellular variant of leiomyoma with frequent scattered macrophages characterized by pale vacuolated and occasionally pigmented cytoplasm (within white circles; 400× magnification, H&E stain).

## Data Availability

Not applicable.
